# Post-stroke fatigue: a scoping review

**DOI:** 10.12688/f1000research.22880.2

**Published:** 2020-08-25

**Authors:** Ghazaleh Aali, Avril Drummond, Roshan das Nair, Farhad Shokraneh

**Affiliations:** 1 Division of Psychiatry and Applied Psychology, University of Nottingham, Nottingham, UK; 2 Institute of Mental Health, Nottinghamshire Healthcare NHS Foundation Trust, Nottingham, UK; 3 Faculty of Medicine and Health Sciences, School of Health Sciences, Queen’s Medical Centre, University of Nottingham, Nottingham, UK; 4 King's Technology Evaluation Centre (KiTEC), London Institute of Healthcare Engineering, School of Biomedical Engineering and Imaging Sciences, Faculty of Life Sciences and Medicine, King's College London, London, UK

**Keywords:** Post-Stroke Fatigue, Scoping Review

## Abstract

**Background**: Post-stroke fatigue (PSF) is one of the most common and frustrating outcomes of stroke. It has a high prevalence and it can persist for many years after stroke. PSF itself contributes to a wider range of undesirable outcomes that affect all aspects of daily life. The aim of this review was to identify and summarise the most recent research on PSF, in order to update the evidence base.

**Methods**: We updated an existing review (Hinkle *et al*. 2017) systematically searching CINAHL, MEDLINE, PsycINFO, and PubMed to cover new research studies between 1 ^st^ March 2016 and the search date (19 ^th^ January 2020). We included interventional and observational research, and clinical practice guidelines that were not covered in the original review. After duplicate removal in EndNote, two reviewers screened the search results in Rayyan, and data from eligible full texts were extracted onto an Excel spreadsheet. Finally, we used RobotReviewer and a human reviewer to assess the risk of bias of randomised trials for this scoping review.

**Results**: We identified 45 records for 30 studies (14 observational, 10 interventional studies, and 6 guidelines). Apart from one, the interventional studies were single-centred, had high risk of bias and small sample size (median 50). They investigated exercise, pharmacotherapy, psychotherapy, education, and light therapy. Observational studies mainly reported the factors related to PSF including co-morbidities, depression and anxiety, quality of life, activities of daily living, stroke severity, medication use and polypharmacy, polymorphism, pain, apathy, limb heaviness, neuroticism, mobility, and thyroid-stimulating hormone. Guidelines either did not report on PSF or, when reported, their recommendations were supported by little or low level of evidence.

**Conclusion**: Although we identified a number of recent studies which have added to our current knowledge on PSF, none are robust enough to change current clinical practice.

## Introduction

Post-stroke fatigue (PSF) has been defined as ‘overwhelming feeling of exhaustion or tiredness’, which is unrelated to exertion, and does not typically improve with rest ^1^. It is one of the most common outcomes of stroke and its prevalence varies between 25% and 85%; however, it is generally accepted that it affects 50% of people after stroke ^2^. PSF is linked to undesirable stroke outcomes and affects patients’ participation in studies, adherence to medication, and effectiveness of rehabilitation ^3^. This has a negative impact on patients’ quality of life and daily life activities ^4– 7^, and also contributes to the burden on family members and carers ^8^.

Although researchers have attempted to explain PSF mechanisms ^9^, its aetiology still remains unclear. This is partly because there are many contributing factors to PSF ^8, 10– 32^, and each research team may focus only on some of the factors to find a route for preventing, treating or managing PSF. Any endeavour to find the most effective intervention in the research literature leads to a collection of heterogeneous interventions from physiotherapy ^33^ and exercise ^34– 38^ to psychotherapy, pharmacotherapy, and recently laser therapy ^39– 41^.

As a systematic effort to review these scattered interventions, a Cochrane review ^42, 43^ compared all the tested PSF treatments to a control group, to standard care, or to each other, through reviewing randomised controlled trials (RCTs). This review concluded that there was insufficient evidence of the efficacy of the tested interventions in trials, and more robust research with adequate sample sizes was required ^42, 43^. Since then, more recent systematic reviews until 2019 have attempted to summarise the evidence of effectiveness of Modafinil, mindfulness training, a traditional Chinese medicine, and smart technologies, but still came to a similar conclusion to that of the Cochrane review in 2015 ^44– 47^.

As a result of such uncertainty, current clinical practice guidelines rely on low levels of evidence, such as expert consensus, to make recommendations for PSF ^48, 49^. However, the efforts to design and test treatments continue, which makes it necessary to keep up-to-date with new research and practice literature.

### Objective

The objective of this review was to identify and summarise the most recent research literature related to PSF in order to update the evidence base. As there was an existing review covering the literature up until 2016 ^50^, we only updated the literature not covered in this review.

## Methods

### Methods from an existing review

In 2017, Hinkle *et al.*, ^50^ published a review covering emerging evidence relating to the management of PSF, up to and including February 2016. Because of the comprehensiveness of this review, we only searched for literature published after 1 ^st^ March 2016. As the search methods of the Hinkle *et al.*, review were not reproducible, and the search strategies and results were not available, we contacted the corresponding author and their librarian on 15 ^th^ October 2019. Since we did not receive a reply, we designed the search methods for the reported databases in order to capture the majority of the literature included in Hinkle *et al.*,’s review.

### Scoping review methods

**Figure 1.**
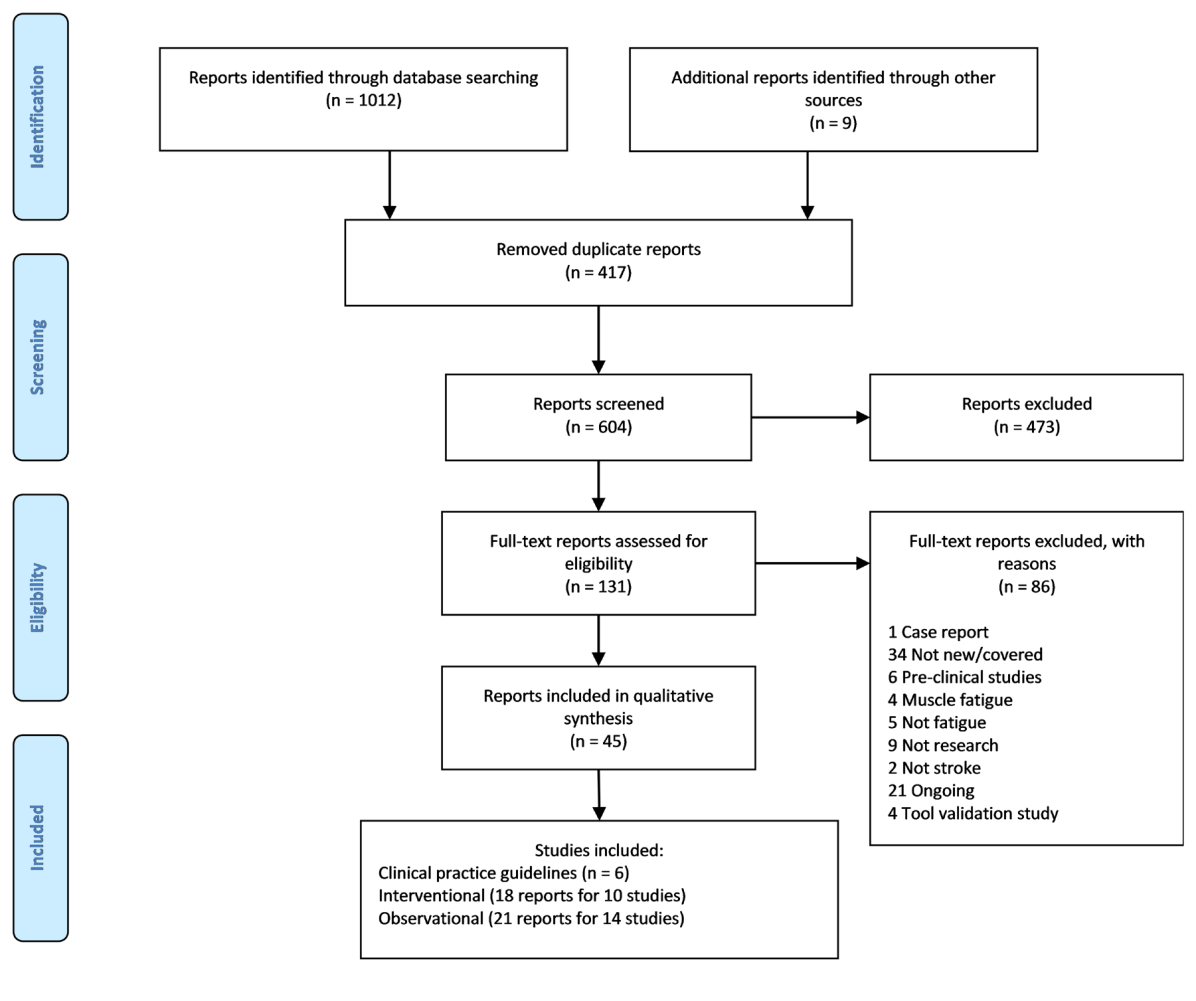
PRISMA flow diagram.

We followed Arksey and O’Malley framework ^51^ for conducting this scoping review. We also used Preferred Reporting Items for Systematic Reviews and Meta-Analyses-Extension for Scoping Reviews(PRISMA-ScR) for reporting ^52^. The relevant PRSIMA-ScR checklist is available as *Extended data* and the flow diagram is reported in the Results section ( Figure 1).

### Search methods

We ran a search to include studies in the English language only, between 1 ^st^ March 2016 and 19 ^th^ January 2020 (search date) in CINAHL via EBSCOhost, MEDLINE via Ovid SP, PubMed (excluding MEDLINE), and PsycINFO via Ovid. There were no limitations to document type (e.g. thesis), study completion status (e.g. ongoing), and publication status (e.g. unpublished) at the search stage. We report the search strategies for all databases in *Extended data*.

### Selection of studies

We imported the search results into EndNote X6 and de-duplicated them based on title, and additionally double-checked the automatically identified duplicates manually. Two reviewers (GA and FS) screened the results independently against the eligibility criteria using Rayyan, which is a recommended screening system ^53^. Discrepancies were resolved through discussions or asking a third reviewer (AD).

Two reviewers (GA and FS) also investigated the full texts of relevant search results against the same criteria involving a third reviewer (AD) in case of disagreement. At full text screening stage, we also investigated the reference lists of the relevant studies to identify additional relevant studies. Since one study may have multiple reports or publications, we kept a record and cited all the reports of a single study to provide a better overview of the new research evidence.

### Eligibility criteria

We included the following studies:

- Studies of adult humans with PSF – any definition of PSF – at any stage of the stroke care continuum;
- Any interventional (clinical trial) or observational (cohort, case-control, and cross-sectional) studies, and clinical practice guidelines;
- Studies reporting findings that had not been included in the previous review;
- Studies included in relevant systematic reviews.

We excluded the following studies:

- Studies with case reports, case series, and qualitative design;
- Studies included in Hinkle *et al.*, or results which repeated the summarised knowledge in that review;
- Studies of pre-clinical nature;
- Clinical studies where fatigue was reported only as a side effect of the treatment;
- Studies focusing on single muscle fatigue or muscle fatigue in general;
- Studies not focusing on fatigue and/or stroke or focusing on heat stroke, athletes’ fatigue or carers’ fatigue;
- Systematic or narrative or review papers;
- Ongoing studies or protocols with no results (listed and cited in this paper for further follow-up);
- Tool validation studies without reporting new findings on PSF.

### Data extraction methods

One reviewer (GA) extracted and entered the data in Excel 2007 and the second reviewer (FS) checked the extracted and entered data against the full text and, if appropriate, corrected or amended the data.

For interventional studies, we extracted PICOS (participants, intervention, comparison, outcomes, and study design) and other data points:

- Study name and year;
- Clinical trial registration number (for further check on selective reporting bias);
- Country of origin;
- Number of centres;
- Patients: Number of patients, type of stroke, time passed after stroke;
- Intervention and controls: name of intervention and duration;
- Primary and secondary outcomes measures in general and fatigue measures in particular, outcome endpoints, and main findings related to PSF;
- Study design (single-arm clinical trial (CT), controlled clinical trial (CCT), or RCT);

For observational studies, we extracted:

- Study name and year;
- Clinical trial registration number (for further check on selective reporting bias);
- Country of origin;
- Number of centres;
- Patients: Number of patients, type of stroke, time passed after stroke;
- Primary and secondary outcomes measures in general and fatigue measures in particular, outcome endpoints, and main findings related to PSF;
- Study design (cohort, case-control, or cross-sectional).

For clinical practice guidelines, we extracted the following data:

- Study name and year;
- Country and organisation who produced the guideline;
- Recommendations on PSF;
- Evidence base reporting the level of evidence or study designs related to the level of evidence.

### Quality assessment methods

We used RobotReviewer for assessing the risk of bias in the four categories of the Cochrane Risk of Bias tool ^54^ for included RCTs. Although this automation system is reliable for checking the risk of bias for certain bias categories ^55, 56^, one of the reviewers (GA) also double-checked and revised RobotReviewer’s assessment and corrected the data where necessary. We also added a ‘selective reporting of outcomes’ category to the list of biases to cover the main biases in Cochrane Risk of Bias tool. Because of ‘scoping’ nature of this review and lack of time and resources, we did not assess the risk of bias for non-RCTs.

### Synthesis methods

We summarised the data from the new relevant literature in tables. We did not proceed to a meta-analysis for fatigue outcomes due to the heterogeneity of studies. We checked if any of the interventional studies considered following the CONSORT ^57^ for reporting RCTs or TIDieR checklist ^58^ to report the components of new interventions.

## Results

The search identified 1021 results. After screening, we included 45 relevant records related to 24 studies and 6 guidelines ( Figure 1).

The characteristics of included interventional studies have been charted in Table 1. The table shows eight RCTs some with multiple reports and one with a follow-up study ^59– 73^, one CCT ^74^, and two single-arm trials ^75, 76^. All studies were based on single centre studies, except for West *et al.*, (2019) which had two centres ^71^. In studies that reported the intervention delivery details, the psychological interventions were delivered individually and face-to-face – rather than online – by psychologists. We also assessed the risk of bias for RCTs and reported the categories of risk in Table 2 with supporting statements in *Extended data*.

**Table 1.** Characteristics of included interventional studies.

Study name	Country	Design	No. of participants	Stroke type	Time after stroke	Interventions	Duration of intervention	Delivered by	Delivery mode
Chen *et al.*, 2016	Taiwan	RCT	41	With CHF	64.95±53.07 D	Inspiratory Muscle Training + TAU v. TAU	10 W (5 D/W)	Respiratory Therapist	NR
Chen *et al.*, 2019	Taiwan	RCT	72	Ischemic	NR	Mind-Body Exercise (Qigong) + TAU v. TAU	10 D	Researchers	Individual
Delva 2019	Ukraine	CCT	39	Ischemic/TIA	≥3 M	Acetylsalicylic Acid (Low Dose v. High Dose)	3 M	NR	NR
Liu *et al.*, 2016	Taiwan	RCT	64	Haemorrhagic /Infraction	≥3 M	Astragalus membranaceus v. Placebo	28 D	NR	NR
Liu *et al.*, 2018	China	RCT	140	NR	NR	Vitamin C v. Wuling	12 W	NR	NR
MIDAS	Australia	RCT	36	NR	≥3 M	Modafinil v. Placebo	6 W	Patients	Individual
		Follow- Up	18/36						
Nguyen *et al.*, 2019	Australia	RCT	15	//	NR	CBT v. TAU	8 W	Psychologists *	Individual
Van Heest *et al.*, 2017	USA	1-Arm CT	49	NR	NR	Fatigue Management Course	6 W	Clinical Psychologist	Individual
West *et al.*, 2019	Denmark	RCT	90	NR	7.6±8.3 (Treatment), 6.0±4.4 (Control) D	Naturalistic Lighting (Artificial Sunlight Spectrum) v. Standard Indoor Lighting	45.3±22.1 (Treatment), 33.7±12.7 (Control) D	NA	Group
Wu *et al.*, 2017	UK	1-Arm CT	12	First/Recurrent	3±24 M	Manualised Psychological Intervention	7 S	Clinical Psychologist	Individual

* Psychologists with doctoral qualifications in clinical neuropsychology.

RCT: Randomised Controlled Trial; CCT: CONTROLLED CLINICAL TRIAL; CT: Clinical Trial; NR: Not Reported; NA: Not Applicable; CHF: Congestive Heart Failure; TIA: Transient Ischaemic Attack; D: Day; W: Week; M: Month; S: Session

**Table 2.** Risk of bias assessed by RobotReviewer and a human reviewer for randomised controlled trials.

Trial	Random sequence generation	Allocation concealment	Blinding of participants and personnel	Blinding of outcome assessment	Selective reporting of outcomes
Chen *et al.*, 2016	?	?	?	+	+
Chen *et al.*, 2019	+	+	?	?	?
Delva 2019	?	?	?	?	?
Liu *et al.*, 2016	+	+	+	+	+
Liu *et al.*, 2018	?	?	?	?	?
MIDAS	+	+	+	+	+
Nguyen *et al.*, 2019	+	+	?	+	?
West *et al.*, 2019	+	+	?	?	+

Question marks in grey cells indicate unclear or high risk of bias and plus signs in white cells show low risk of bias.

Most of the interventional studies have a medium to high risk of bias. Table 2 shows only two studies in white cells (indicating low risk of bias) but both have small sample size consisting of 34 (MIDAS study ^63– 68^) and 64 randomised patients ^61^ respectively.

We identified 14 observational studies of which half had a prospective cohort design ^77– 90^ and the other half were cross-sectional surveys ^91– 97^. Three cross-sectional surveys were embedded within cohort studies ^91, 94, 97^. Only one of the studies (NotFAST) had a follow-up report ^81– 85^. Details of all studies are reported in Table 3 as well as the *Extended data*.

**Table 3.** Characteristics of included observational studies.

Study name	Country	No. of centres	Design	No. of participants	Stroke type	Time after stroke **
ARCOS-IV	New Zealand	4	Cross-Sectional (in Cohort)	256/2096 *	First,Ischemic,Haemorrhagic,Undetermined	4 Y
Blomgren *et al.*, 2019	Sweden	1	Cohort	296/411	First,Recurrent, Ischemic	7 Y
Chen *et al.*, 2018	USA	1	Cohort	128/203	Ischemic, Haemorrhagic	6 M
Choi-Kwon *et al.*, 2017a	South Korea	1	Cross-Sectional	373/469	Ischemic	3 M
Choi-Kwon *et al.*, 2017b	South Korea	1	Cohort	364/508	Ischemic	12 M
Douven *et al.*, 2017	Netherlands	2	Cohort	243/250	First, Ischemic	3, 6, 12 M
Kuppuswamy *et al.*, 2016	UK	3	Cross-Sectional	69	First	56.81±63 M
LAS-1	Sweden	1	Cross-Sectional (in Cohort)	349	NR	6 Y
Lau *et al.*, 2017	Hong Kong	1	Cross-Sectional	191	Ischemic	3 M
MacIntosh *et al.*, 2017	Canada	4	Cross-Sectional	335	Ischemic, Haemorrhagic	Within 6 M
NotFAST	UK	4	Cohort	268/371	First	4-6 W
			Follow-Up	263/371		6 M
STROKDEM	France	4	Cohort	153/179	Ischemic, Haemorrhagic	6 M
van Rijsbergen *et al.*, 2019	Netherlands	1	Cross-Sectional (in Cohort)	208	First, Ischemic, Haemorrhagic, Recurrent	3.3±0.5 M
Wang *et al.*, 2018	China	1	Cohort	634/703	Ischemic	Within 3 D

NR: Not Reported; Y: Year; M: Month; W: Week; D: Day

*For cohort studies, the left number shows the number of participants who finished follow-up, and the right number is the number of participants who started and took part in the study; for cross-sectional studies within cohort studies, the left number shows the number of participants in cross-sectional study and the right number is the number of participants in cohort study. ** Sometimes reported as time period and sometimes as mean and standard deviation across the studies.

Table 4 summarises the main finding of each interventional study all of which either have high risk of bias or small sample size. Such limitations make it hard to transfer the research findings to practice.

**Table 4.** Descriptive summary of findings from included interventional studies.

Study Name	Fatigue measure	Endpoint	Main post-stroke fatigue finding *
Chen *et al.*, 2016	Secondary: FAS	W 10	There were no significant changes from baseline in FAS in either group (intervention: p=0.218; control: p=0.475; change between groups: p=0.198).
Chen *et al.*, 2019	Fatigue VAS	D 5, 10	Fatigue was not significantly associated with change in quality of life (β = −0.21,95% CI [−0.73~;0.31],p=0.42) and was not different in two groups.
Delva, 2019	FAS	D 3, M 1, 3	The use of aspirin in high dose during 3 months with PSF diagnosis within the first days post-stroke is associated with decreasing of fatigue intensity due to FAS and modifying of post-stroke inflammatory response (р<0.05).
Liu *et al.*, 2016	Primary: BFI	D28±5, 84±5	Astragalus membranaceus group had improved fatigue in visit 1–28± 5 days – post- therapy (p=0.01) and in follow-up in 84 ± 5 days post-therapy (p=0.05).
Liu *et al.*, 2018	Barthel Index	W 12	Wuling can inhibit the release rate of inflammatory factor, reduce the expression level of related inflammatory factors and improve PSF (p<0.05).
MIDAS	Primary: MFI	W 6, 7	Modafinil group reported decrease in fatigue (β = −7.38, 95% CI [−21.76; −2.99],p<0.001) and improvement in quality of life (β = 11.81, 95% CI [2.31; 21.31], p=0.0148).
		M 12	MFI and quality of life at baseline and their changes during treatment were correlated (β = −1.975, 95%CI [−3.082; −0.869], p < 0.001). Five of the patients who continued taking daily modafinil demonstrated 33–38 point simprovement in MFI compared to baseline.
Nguyen *et al.*, 2019	Primary: FSS	M 2, 4	CBT group demonstrated reduced fatigue relative to TAU post-therapy (β = 1.74,95%CI [0.70; 2.77],effect size (η2)=.52)and two months post-therapy (β =1.92, 95% CI [0.24; 3.60],effect size (η2)=.36).
Van Heest *et al.*, 2017	Primary: FACIT- Fatigue	W 6, 12	Participants showed reductions in fatigue at post-test (p<0.001; effect size (Cohen’s d) = 21.19) and maintained it at follow-up (p=0.315; effect size (Cohen’s d) = 0.23).
West *et al.*, 2019	Primary: MFI	Discharge	At discharge, patients from the naturalistic light group experienced less fatigue than the indoor light group (diff = –20.6%, 95% CI [–35.0%; –3.0%],p = 0.025).
Wu *et al.*, 2017	Secondary: FAS	S 6, 1, M 3	Fatigue decreased post-treatment (mean difference=4.8, 95% CI [-2.1; 11.6], p = 0.15), in one-month assessment (mean difference=7.0, 95% CI [-0.8; 14.8], p = 0.07), and in three-month assessment (mean difference=9.3, 95% CI [1.4; 17.1]; p = 0.03).

FAS: Fatigue Assessment Scale; VAS: Visual Analogue Scale; BFI: Brief Fatigue Index; MFI: Multidimensional Fatigue Inventory; FSS: Fatigue Severity Scale; FACIT: Functional Assessment of Chronic Illness Therapy; W: Weeks; D: Day; M: Month; S: Session; TAU: Treatment As Usual; CBT: Cognitive Behavioural Therapy.

*Grey cells contain findings from low risk studies; however they have small sample size. We reported the data as reported in the original report.

The majority of observational studies investigated factors related to PSF including co-morbidities, physical and mental outcomes, illness characteristics, characteristics of interventions, and biomarkers ( Table 5).

**Table 5.** Descriptive summary of findings from included observational studies.

Study name	Fatigue measure	Post-stroke fatigue finding
ARCOS-IV	FSS	Having hypertension, diabetes mellitus, and arrhythmia at the time of stroke were associated with increased PSF.
Blomgren *et al.*, 2019	FIS	Fatigue was independently explanatory of worse outcome on FAI summary score and domestic chores.
Chen *et al.*, 2018	FACIT- Fatigue	Early PSF appears to be largely attributable to stroke severity, while chronic fatigue occurs in the setting of medical co-morbidities and medication use.
Choi-Kwon *et al.*, 2017a	FSS	Of the 6 polymorphisms examined, only one marker, that is, low-activity Monoamine Oxidase A was associated with PSF in female patients.
Choi-Kwon *et al.*, 2017b	FSS	Musculoskeletal pain and central post-stroke pain was related to fatigue.
Douven *et al.*, 2017	FSS	No association between apathy and fatigue was found at baseline and no interaction with time was found. Change in fatigue from baseline to 12-month follow-up was associated with change in depression and with change in apathy. Bidirectional associations were found between PSF and PSD.
Kuppuswamy *et al.*, 2016	FSS	Those with high perceived limb heaviness also reported significantly higher levels of fatigue than those with no perceived limb heaviness, but there was no difference in weakness between the 2 groups.
LAS-1	FSS	In almost all Stroke Impact Scale domains the odds for PSF were higher in persons with a higher perceived impact. Fatigue is still present in one-third of persons six years after stroke onset.
Lau *et al.*, 2017	FAS	Fatigue severity positively correlated with NEO Five-Factor Inventory neuroticism scores.
MacIntosh *et al.*, 2017	FAS	Fatigue and depressive symptoms are related distinctly to cognitive and mobility impairments post-stroke. Fatigue was associated with poorer lower limb motor function, and with cognition indirectly via depressive symptoms.
NotFAST	FSS of FAI	Pre-stroke fatigue, having a spouse/partner, lower Rivermead Mobility Index score, and higher scores on both the Brief Assessment Schedule Depression Cards and Beck Anxiety Index were independently associated with PSF.Of those reporting fatigue initially 69% continued to report fatigue in follow-up. New PSF cases were reported by 38%. Lower Nottingham Extended Activities of Daily Living scores and higher Beck Anxiety Index scores were independently associated with fatigue at six months.
STROKDEM	CFS	Medication use was not a PSF predictor; however, polypharmacy increased PSF severity.
van Rijsbergen *et al.*, 2019	FAS	Fatigue was associated with CLCE scores, independent of demographic, cognitive performance and stroke-related covariates. After including personality traits and coping styles in the model, independent associations with CLCE scores were found for fatigue and neuroticism.
Wang *et al.*, 2018	FSS	The serum levels of thyroid-stimulating hormone were inversely associated with the risk of PSF in both the acute phase and at follow-up. Thyroid function profiles may be predictor of PSF after acute ischemic stroke.

FSS: Fatigue Severity Scale; FIS: Fatigue Impact Scale; FACIT: Functional Assessment of Chronic Illness Therapy; FAS: Fatigue Assessment Scale; FAI: Fatigue Assessment Inventory; PSD: Post-Stroke Depression; CFS: Chalder Fatigue Scale; CLCE: Checklist for Cognitive and Emotional consequences following stroke

We identified six recent guidelines from three English-speaking countries including the UK ^49^ and two North American countries (one from Canada ^48^ and four from the USA ^98– 101^). Among these, the Canadian guideline was the most recent and the only one with comprehensive recommendations on PSF. The UK guideline will be updated in 2021. Half of these guidelines, that is, all those from USA, have not provided specific recommendations on PSF, as reported in Table 6. In almost all the guidelines, the reliance on ‘experts’ consensus’ is apparent because of the limited evidence base for PSF ( Table 6).

**Table 6.** Descriptive summary of included clinical practice guidelines.

Citation	Country- Organisation	Recommendation	Evidence base
Braun *et al.*, 2016	USA-AHA/ASA	None	NR
Lanctot *et al.*, 2019	Canada-CSBPR	See pages 15–16 of guideline.	RCT, CCT, CT, Consensus
NICE 2017	UK-NICE	Assess the person for mental and physical factors that may contribute to fatigue. Treat any reversible causes or exacerbating factors. Provide the person and their family/carers with information and help in anticipating and managing fatigue such as daily routines, modified tasks which balance activity and rest, planned exercise schedules, and sleep hygiene.	Cochrane SR, SR, Consensus
Peberdy *et al.*, 2017	USA-AHA	None	NR
VA-DoD 2019	USA-VA/DoD	None	NR
Winstein *et al.*, 2016	USA-AHA/ASA	An RCT compared a multi-component cognitive therapy + graded activity training versus cognitive therapy for 12 weeks and showed that the multi-component therapy is better than the cognitive therapy in reducing fatigue and improving physical endurance … myths about exercise being unsafe, causing another stroke, or increasingfatigueshould be dispelled during rehabilitation … evidence is limited, many clinicians advise that for individuals who want to return to work, a tailored assessment of cognitive, perception, physical, and motor abilities can be performed to determine readiness and the needed accommodations to return to work based on individual’s needs and capabilities for the specified job situation. The assessment may include executive functions, high-level oral and written communication, and fatigue. Once performance under the best conditions has been assessed, further assessment under conditions of fatigue and stress may be useful to mimic potential job situations.	RCT

AHA: American Heart Association; ASA: American Stroke Association; VA/DoD: Department of Veterans Affairs/Department of Defense; NICE: National Institute for Health and Care Excellence; CSBPR: Canadian Stroke Best Practice Recommendations by Management of Mood, Cognition and Fatigue Following Stroke Best Practice Writing Group/Heart & Stroke Canadian Stroke Best Practices and Quality Advisory Committee/Canadian Stroke Consortium; NR: Not Reported; RCT: Randomised Controlled Trial; CCT: Controlled Clinical Trial; CT: Clinical Trial; SR: Systematic Review.

## Discussion

We conducted this review to identify and summarise the most recent research studies on PSF since Hinkle *et al.*,’s review (2017) ^50^. We therefore documented the interventional and observational research and clinical practice guidelines since March 2016. However, there were some key contributors to the weak evidence base: (i) recording and reporting only some contributing factors to PSF in observational studies, (ii) the heterogeneity of designed interventions, (iii) high risk of bias, (iv) small sample size in interventional studies, and (v) variety of outcome measures in both observational and clinical studies. This, in turn, is reflected in the quality of the clinical recommendations for PSF.

Despite the high prevalence of PSF ^2^ and its obvious effects on treatment adherence ^102^, in practice, only half of recent stroke guidelines have clinical recommendations on PSF. Of those that do, two guidelines provide only brief recommendations, and only one provides comprehensive recommendations, but these are based on low levels of evidence ^48^. The weak evidence base and the need to rely on expert consensus is likely to be the main reason that PSF is generally not covered in the guidelines.

The dominance of single-centred interventional studies with small sample sizes and interventions delivered within a 12-week period may be the reasons for absence of follow-up studies. MIDAS (interventional) ^63– 65^ and NotFAST (observational) ^81– 85^ are the only recent studies with novel and potentially long-term findings with larger sample size (in case of MIDAS 2) ^103^ or with the intention to design an intervention (NotFAST2) ^104^.

While the observational studies reported the type of stroke, the interventional studies did not include this important data, which makes it difficult to summarise studies. Most of participants entered the interventional studies three-months after stroke. This is likely to be due to a number of reasons; for example, fatigue is not recognised immediately after a stroke, some studies want to ensure that participants have a continuous fatigue, and there is competition for recruitment in the early stages to more acute trials. However, one issue worth considering is whether the construct of PSF holds for fatigue experienced in research participants recruited years after their stroke, and whether this fatigue is a function of other issues. Future systematic reviews could address this issue by conducting sensitivity analyses comparing studies that include participants many years after their stroke with those including participants immediately after their stroke.

The variety of the interventions tested in studies and trials underlines the complexity of PSF and is an indication to researchers that future interventions will probably need to target multiple aspects of fatigue. While current reporting practice of interventions in RCTs included in our review is of concern (none followed TIDieR and two followed CONSORT), future studies should consider following reporting guidelines such as CONSORT and TIDieR for interventional studies, STROBE for observational studies, and RIGHT ^105^, AGREE ^106^, or CheckUP ^107^ for clinical practice guidelines. In addition, harmonisaton of studies requires standard international guidelines regarding outcome measurements and time points for measuring PSF in a standard way to create a homogenous and collective body of evidence.

Among the observational studies, the population-based study from the stroke register in New Zealand ^91^ and Sweden ^77^ provides valuable insights about the link between co-morbidities and increased PSF in long-term (4–7 years). This, and other similar register-based studies, represent the added value of having high-quality data in health system databases for long-term observational and register-based studies ^108^.

Psychologists delivered the psychotherapies in RCTs to individual patients and there was no intervention using online platforms as the media of delivery. This may be due to a number of reasons: it is usual to test the efficacy of an intervention face to face before moving to another medium; participants with stroke may have other problems which mean it is more difficult to deliver treatments online, e.g. communication issue and cognitive problems. Online delivery of such interventions is becoming more common in some clinical services, and new research is emerging ^109^. Therefore, future reviews may wish to consider the impact of such interventions delivered online. 

Fatigue Severity Scale (FSS) was the main outcome measure for PSF in observational studies, whereas Fatigue Assessment Scale (FAS) was used more frequently than other measures in interventional studies. Bearing in mind that both these PSF measurement scales are valid and reliable, the main reason that the FSS has been used more frequently is probably because it is now seen as a way to compare different studies: in simple terms, researchers use it because other researchers have used it. It is also relatively straight forward to complete.

Only one of the observational studies and half of the interventional studies were registered in clinical trial registers, with the remaining unregistered trials potentially introducing bias in selective reporting of outcomes ^110, 111^. One of the interventional studies was registered retrospectively with potential for the same bias ^69, 70^.

### Limitations and strengths

It is possible that we overlooked studies that did not report PSF in the searchable part of the paper or if the report was not indexed in the searched databases. In such cases, we invite readers of this review to contact us or comment on the paper online.

Due to the challenges we faced in identifying the search strategies in the previous review when conducting this update, we have explicitly documented our search criteria and strategies so that this review can be easily repeated and updated by future researchers. We feel this is a key strength of this review. We also feel that the use of automation tools such as Rayyan and RobotReviewer for this evidence synthesis was a strength in terms of saving time and other resources while still maintaining the quality of the review. Finally involving a multi-disciplinary team of clinicians and methodologists (information specialist and systematic reviewer) allowed us to consider both the clinical and methodological aspects of the studies in this review.

## Conclusion

The current trend of research on PSF shows the continued importance of this topic globally. Our review identified a weak evidence base that highlights the need for more research that could have the following characteristics: I) studies to design and test multi-component interventions for PSF; and II) Robust RCTs with adequate sample sizes to produce the evidence for recommendations in guidelines. From our current knowledge on PSF, none of the recent studies are robust enough to change current clinical practice.

## Data availability

### Underlying data

Open Science Framework: Post-Stroke Fatigue: A Scoping Review, https://doi.org/10.17605/OSF.IO/XJKCS ^112^.

Registration DOI: https://doi.org/10.17605/OSF.IO/XJKCS

This project contains the following underlying data:

- Extracted data from included studies

### Extended data

Open Science Framework: Post-Stroke Fatigue: A Scoping Review, https://doi.org/10.17605/OSF.IO/XJKCS ^112^.

This project contains the following extended data:

- Full search strategies
- Risk of bias assessment

### Reporting guidelines

Open Science Framework: PRISMA-ScR checklist for ‘Post-stroke fatigue: a scoping review’, https://doi.org/10.17605/OSF.IO/XJKCS ^112^.

Data are available under the terms of the Creative Commons Attribution 4.0 International license (CC-BY 4.0).
