# Supplementary material for: Post-stroke fatigue: a scoping review
Source: F1000Res. 2020 Aug 25;9:242. Originally published 2020 Apr 7. [Version 2] doi: 10.12688/f1000research.22880.2 (PMC7468563; doi:10.12688/f1000research.22880.2)
Supplement: Peer review history

# Peer review: Reviewer response for version 2

**DOI:** 10.5256/f1000research.27636.r70217

**Published:** 2020-09-02

**Authors:** Carina Persson

**Affiliations:** 1 Department of Clinical Neuroscience, Rehabilitation Medicine, Institute of Neuroscience and Physiology, Sahlgrenska Academy, University of Gothenburg, Gothenburg, Sweden

The authors perfectly solved or clarified all the issues that I raised. I would like to congratulate them on this interesting paper.

Are the rationale for, and objectives of, the Systematic Review clearly stated?

Yes

Is the statistical analysis and its interpretation appropriate?

Not applicable

Are sufficient details of the methods and analysis provided to allow replication by others?

Yes

Are the conclusions drawn adequately supported by the results presented in the review?

Yes

Reviewer Expertise:

NA

I confirm that I have read this submission and believe that I have an appropriate level of expertise to confirm that it is of an acceptable scientific standard.

# Peer review: Reviewer response for version 1

**DOI:** 10.5256/f1000research.25259.r62109

**Published:** 2020-05-04

**Authors:** Nicola Hancock

**Affiliations:** 1 School of Health Sciences, University of East Anglia, Norwich, UK

Thank you for the invitation to review this interesting scoping review, focussing on an important area of current research highly relevant to multiple aspects of life after stroke- that of Post-Stroke Fatigue, PSF.

 The current review updates the work of Hinkle *et al. *2016, and the rationale for doing so is clearly stated by the authorship team. That the review here generated a further 24 studies (and six sets of guidelines including PSF) since 2016 further demonstrates the growing impetus of work in this area. The objective stated is rather non-specific, but this is acceptable in such a scoping review that serves as a summary of recent evidence. It is unsurprising that this review concludes that further research in this area is required, and the authors make relevant suggestions as to the characteristics of future research that are clearly based on the findings of this review.

 The review is clearly written, and the methods used and ensuing findings have been reported with transparency and considerable attention to detail. Search strategies are available via an embedded link. This paper makes a very useful contribution and provides a foundation for further work.

 The following minor comments and suggestions may be of use to the authors:

- The use of the word ‘following’ in the opening two section headings of the methods might be worth reconsidering, readability of this phrase in a title is challenging (page 3).
- In Figure 1, suggest clarifying ’34 not new/covered’- does this mean 34 records removed as covered in the previous review? (page 4).
- Section on data extracted from clinical practice guidelines, please clarify ‘study name and year’- does this refer to the name and year of the guidelines or the included studies from which the guidelines was written? (page 5).
- Was any specific tool used to assess risk of bias in the non-randomised studies? If so, this should be stated in the methods text. If not, some justification would be helpful.
- In table 3, the final column is not entirely clear- is this mean time after stroke onset for included participants? Simple clarification in the column heading or legend would address this (page 7/8).
- Suggest rephrasing ‘probably the most effective interventions’ to deliver a clearer message here (page 9).
- In the discussion, the section on online platforms is somewhat unexpected- possible delivery via online mechanisms does not seem to have arisen prior to this point, though I apologise if I have missed this. Perhaps a line to place this paragraph in context might help the interpretation here? (page 10).
- As the authors focus on possible reasons for the use of the Fatigue Severity Score (FSS) in one section of the discussion, it might be helpful to include a line about the validity/reliability of this measure at this point.
- There are a few very minor typographical and grammatical errors.

Are the rationale for, and objectives of, the Systematic Review clearly stated?

Yes

Is the statistical analysis and its interpretation appropriate?

Not applicable

Are sufficient details of the methods and analysis provided to allow replication by others?

Yes

Are the conclusions drawn adequately supported by the results presented in the review?

Yes

Reviewer Expertise:

Stroke rehabilitation

I confirm that I have read this submission and believe that I have an appropriate level of expertise to confirm that it is of an acceptable scientific standard.

# Response

**Published:** 2020-06-23

**Authors:** Ghazaleh Aali

**Affiliations:** Division of Psychiatry and Applied Psychology, University of Nottingham, UK

We thank the expert reviewer for spending time on our review and for providing helpful comments. We have made amendments to the manuscript based on these comments.

**Comment**: The use of the word 'following' in the opening two section headings of the methods might be worth reconsidering, readability of this phrase in a title is challenging (page 3).

**Reply**:Thank you. We have now changed these to 'Methods from an existing review' and 'Scoping review methods'.

**Comment**: In Figure 1, suggest clarifying '34 not new/covered'- does these mean 34 records removed as covered in the previous review (page 4)?

**Reply**:We have now clarified this in the text.We excluded  studies that had been covered in the previous review. We also excluded the new studies that were not covered in the previous review if they repeated the findings reported in the previous reviews. We included new studies that were repeating findings from the previous review if they had a larger sample size or a new factor or intervention so were adding to the existing knowledge. This is compatible with the 'scoping' element of this review.

**Comment**: Section on data extracted from clinical practice guidelines, please clarify 'study name and year'- does this refer to the name and year of the guidelines or the included studies from which the guidelines was written (page 5).

**Reply**: The 'Citation'column refers to the reference publication and publication date of the guideline.

**Comment**: Was any specific tool used to assess risk of bias in the non-randomised studies? If so, this should be stated in the methods text. If not, some justification would be helpful.

**Reply**:Assessing risk of bias is not usually a feature of scoping reviews. However, because of availability of the automation tool (i.e. RobotReviewer) for RCTs and to provide additional training to two PhD students who were involved in this review, we added risk of bias assessment only for the RCTs. We did not assess non-RCTs for risk of bias because our resources were limited. We have now addressed this in the text: "Because of the ‘scoping’ nature of this review and lack of time and resources, we did not assess the risk of bias for non-RCTs."

**Comment**: In Table 3, the final column is not entirely clear- is this mean time after stroke onset for included participants? Simple clarification in the column heading or legend would address this (page 7/8).

**Reply**:It is both. Because of the lack of standards in reporting these data, some studies reported time period after stroke and some reported only mean and standard deviation.We have now detailed this under the table.

**Comment**: Suggest rephrasing 'probably the most effective interventions' to deliver a clearer message here (page 9).

**Reply**: We have changed 'the most effective' to 'the future'.

**Comments**: In the discussion, the section on online platforms is somewhat unexpected- possible delivery via online mechanisms does not seem to have arisen prior to this point, though I apologise if I have missed this. Perhaps a line to place this paragraph in context might help the interpretation here (page 10)?

**Reply**:Thank you. In the second paragraph of the Results we note that the delivery was mostly face-to-face and individually rather than online.

**Comment**: As the authors focus on possible reasons for the use of the Fatigue Severity Score (FSS) in one section of the discussion, it might be helpful to include a line about the validity/reliability of this measure at this point.

**Reply**:We have added a sentence about validity/reliability: "Bearing in mind that both these PSF measurement scales are valid and reliable…". We therefore tried to raise the other 'possible' reasons for the frequent use of this specific scale in this field.

**Comment**: There are a few very minor typographical and grammatical errors.

**Reply**: Thank you. We have now addressed these and apologise for overlooking these.

# Peer review: Reviewer response for version 1

**DOI:** 10.5256/f1000research.25259.r62111

**Published:** 2020-04-22

**Authors:** Carina Persson

**Affiliations:** 1 Department of Clinical Neuroscience, Rehabilitation Medicine, Institute of Neuroscience and Physiology, Sahlgrenska Academy, University of Gothenburg, Gothenburg, Sweden

I found this scoping review very exciting to read since it addresses a very important issue that needs to be managed daily in post-stroke rehabilitation. I believe that the interest in post-stroke fatigue is great from both researchers and clinically active colleagues. I am positive to and welcome this type of “game-changer” by a transparent scientific publishing.

 The title identifies the manuscript as a scoping review and reflects the population (individuals post-stroke) and the construct (post-stroke fatigue). The abstract is presented with a clear structure, including background, methods, results and conclusion, of which all parts relate to the aim of the study. The rational for the review is described; there is insufficient evidence of the efficacy of the interventions related to post-stroke fatigue in conducted trials and more robust research with adequate sample sizes is required. In addition, it was time to make an update. The objective is well described, to identify and summarise the most recent research related to post-stroke fatigue in order to update the evidence base. The update covers new research studies between 1 ^st ^March 2016 and 19 ^th^ January 2020. The search strategies for the databases are reported in a separate link ( *Extended data)*. In the results, data related to the objective of the study is presented with a clear structure in six tables. These tables include characteristics of, and summary of the findings from, the included interventional and observational studies, risk of bias and a summary of included guidelines. Due to the heterogeneity of studies, no meta-analysis for fatigue outcomes was performed. The interventional studies were limited by either having high risk of bias or small sample size. Consequently, transferring the research findings to practice was hard.

 The current manuscript is well written and easy to follow. It is clear that you have put in a lot of effort and made a great job. Even so, I have following minor comments/suggestions that could possibly improve your article:

**Method: **

- As written, I am uncertain about limitations to language. According to the manuscript, there were no limitations to language. However, in the *Extended data*, English language seems to be a limitation in the search strategies. Please, clarify so there is no doubt about this.
- Figure 1: Please, consider clarifying the number of excluded reports per each specified criterion regarding the 473 excluded reports; no clarification (in numbers) has been made for them as for the 86-excluded full-text reports.
- Figure 1: Please, give the readers more information about the nine additional sources. Which was your strategy, how did you found them? Did all arrived from the other articles´ reference lists?

**Results:**

- Are MIDAS (presented in Table 1), and ARCOS-IV and LAS-1 (presented in Table 3) examples of studies not yet published?
- (In Table 1, the fourth column, there is space available (two rows are already used) for you to write “Number of participants” or “No.” instead of using the symbol “#”.)
- Table 2: I like the use of different colours, and red and green are instantaneous to understand. However, in color blindness, the most common difficulty is to distinguish between red and green. Perhaps you can choose another colour combination. In the online version of Robot Reviewer report, I think that the table Risk of bias has a more easy to read layout than Table 2 in the main manuscript.
- (Table 3: Consider using “Number” or  ”Number of” or “No.” or “No. of” instead of “#” in the third and the fifth column?)
- Table 3, sixth column, “Stroke type”: Please, review the use of “/”. Should you use “and”, “,” or delete the “/” somewhere? Regarding Kuppuswamy and NotFAST: “First”, but which stroke type?
- (Table 4, fourth column: I suggest you to use “Post-stroke fatigue finding” instead of the use of the abbreviation “PSF finding”. Avoiding unnecessary abbreviations makes reading easier.)
- (In Table 5, consider if you should specify “Finding” to “Post-stroke fatigue finding” in the light blue heading (in line with the selected sub-heading in Table 4).)
- In the data commentary related to Table 6, you state that the Canadian guideline was the only one with comprehensive recommendations on post-stroke fatigue. In Table 6, under the heading “Recommendation” the reader is referred to page 15-16 of the guideline. I suggest that you present vital parts of the content in Table 6, in addition to this reference.
- I lack information on age and gender of the patients in the different studies from which the review is based. Is it possible to add this information?
- Is it possible to give the reader even more specific information on which quantitative measures the post-stroke fatigue findings are based? (Although the level of significance is arbitrary set and statistically significance results does not need to be clinically meaningful for the patients.)

**Discussion:**

- I suggest a sentence about time-dependent concerns regarding the construct (post-stroke fatigue) in the research studies conducted years after stroke and its potential significance for validity.
- International recommendations regarding outcome measures related to, and time points for measuring, post-stroke fatigue in research studies on recovery after stroke would probably reduce the heterogeneity of studies and facilitate further summarises and updates. You could possibly include that post-stroke fatigue might be an issue for future Stroke Recovery and Rehabilitation Roundtable (SRRR) work.
- You have a separate paragraph regarding limitations. Have you thought about including a sentence related to strengths of your study, in a paragraph in close proximity to “Limitations”?

Are the rationale for, and objectives of, the Systematic Review clearly stated?

Yes

Is the statistical analysis and its interpretation appropriate?

Not applicable

Are sufficient details of the methods and analysis provided to allow replication by others?

Yes

Are the conclusions drawn adequately supported by the results presented in the review?

Yes

Reviewer Expertise:

NA

I confirm that I have read this submission and believe that I have an appropriate level of expertise to confirm that it is of an acceptable scientific standard, however I have significant reservations, as outlined above.

# Response

**Published:** 2020-06-23

**Authors:** Ghazaleh Aali

**Affiliations:** Division of Psychiatry and Applied Psychology, University of Nottingham, UK

We are grateful to the expert reviewer for their interest in the topic and for spending time in reviewing this manuscript. We value their positive and thorough comments and have revised the manuscript accordingly.

**Method**

**Comment**: As written, I am uncertain about limitations to language. According to the manuscript, there were no limitations to language. However, in the extended data, English language seems to be a limitation in the search strategies. Please, clarify so there is no doubt about this.

**Reply**: Thank you for this comment. We initially intended to have no language restrictions but, as we followed the methods from an existing scoping review, we needed to apply a language limitation. This has now been corrected to: "We ran a search to include studies in English language only…".

**Comment**: Figure 1: Please, consider clarifying the number of excluded reports per each specified criterion regarding the 473 excluded reports; no clarification (in numbers) has been made for them as for the 86-excluded full-text reports.

**Reply**: We followed the PRISMA reported guideline and flow diagram which does not specify recording or reporting detailed reasons for exclusion at the Title/Abstract Screening step (Liberati et al. 2009; DOI  10.1136/bmj.b2700); however, as it is mandatory to report the reasons for exclusion in the Full Text Screening step,  we have done this.

**Comment**: Figure 1: Please, give the readers more information about the nine additional sources. Which was your strategy, how did you found them? Did all arrive from the other articles' reference lists?

**Reply**: We have now included this statement under the Selection of studies section: "we also investigated the reference lists of the relevant studies to identify additional relevant studies ".

**Results**

**Comment**: Are MIDAS (presented in Table 1), and ARCOS-IV and LAS-1 (presented in Table 3) examples of studies not yet published?

**Reply**: No. These studies were completed and have reported their results, so they met our eligibility criteria. If a study was 'ongoing' at the time of our review, we did not report them in the table because "Ongoing studies or protocols with no results"was one of our exclusion criteria. If a study had no specific name, we used the last name of the first author and the year of publication as the study name in the tables.

**Comment**: In Table 1, the fourth column, there is space available (two rows are already used) for you to write "Number of participants" or "No." instead of using the symbol "#".

**Reply**:We have corrected this now to "No. of".

**Comment**: Table 2: I like the use of different colours, and red and green are instantaneous to understand. However, in colour blindness, the most common difficulty is to distinguish between red and green. Perhaps you can choose another colour combination. In the online version of Robot Reviewer report, I think that the table Risk of bias has a more easy to read layout than Table 2 in the main manuscript.

**Reply**: We followed Cochrane's Risk of Bias tool and therefore also followed their reporting method. However we recognise that some of our readers might be colour-blind and so we are using plus signs and question marks in addition to the colour coding. We have also now changed the colours from red/green to grey/white.

**Comment**: Table 3: Consider using "Number" or "Number of" or "No." or "No. of" instead of "#" in the third and the fifth column?

**Reply**:We have corrected these to "No. of".

**Comment**: Table 3, sixth column, "Stroke type": Please, review the use of "/". Should you use "and", "," or delete the "/" somewhere? Regarding Kuppuswamy and NotFAST: "First", but which stroke type?

**Reply**: We replaced "/" with" ,". We used type of stroke as reported in the studies. Thus, although 'First' does not refer to a specific stroke type, this was what was reported and therefore what we presented under 'type'. Since this is a scoping review, we did not contact specific researchers to clarify these details.

**Comment**: Table 4, fourth column: I suggest you to use "Post-stroke fatigue finding" instead of the use of the abbreviation "PSF finding". Avoiding unnecessary abbreviations makes reading easier.

**Reply**: We have now corrected this to 'post-stroke fatigue'.

**Comment**: In Table 5, consider if you should specify "Finding" to "Post-stroke fatigue finding" in the light blue heading (in line with the selected sub-heading in Table 4).

**Reply**: We have now added 'post-stroke fatigue'.

**Comment**: In the data commentary related to Table 6, you state that the Canadian guideline was the only one with comprehensive recommendations on post-stroke fatigue. In Table 6, under the heading "Recommendation" the reader is referred to page 15-16 of the guideline. I suggest that you present vital parts of the content in Table 6, in addition to this reference.

**Reply**: We did consider this approach initially. However, for the following reasons we decided not to report the text for this guideline: 1. there are two pages of content that could be paraphrased and summarized into the table but because of the volume of the content (even after summarizing and paraphrasing) it would require official copyright permission from the publisher of the guideline. Aside from the process of obtaining such permission, this will require payment to the publisher which is not included within our grant. 2. We found the content of these two pages relevant, well-written, and important, and we have consequently intentionally referred the reader to this source, rather than paraphrasing and losing important detail.

**Comment**: I lack information on age and gender of the patients in the different studies from which the review is based. Is it possible to add this information?

**Reply**: If it is not reported in our review, it means that this information was missing from the primary study. Since this is a scoping review, we did not consider contacting each researcher separately. However, we have highlighted the fact than even very basic but important demographic information has not been reported by researchers of the primary studies. Not including key demographic information is an important issue to highlight, because it needs to be addressed in future studies

**Comment**: Is it possible to give the reader even more specific information on which quantitative measures the post-stroke fatigue findings are based? (Although the level of significance is arbitrary set and statistically significance results does not need to be clinically meaningful for the patients.

**Reply**: Reporting/calculating effect sizes is not routine practice or part of reporting guidelines for conducting scoping reviews. However, we thought this revision could add to the value of the interventional studies so we reported these statistics in Table 4. Since this is not a systematic review, we did not consider contacting the researchers for complete information and we did not calculate or combine the effect sizes. Instead we reported the statistics as they were reported in the original studies.

**Discussion**

**Comment**: I suggest a sentence about time-dependent concerns regarding the construct (post-stroke fatigue) in the research studies conducted years after stroke and its potential significance for validity.

**Reply**:

 Thank you. We have added the following: "One issue worth considering is whether the construct of PSF holds for fatigue experienced in research participants recruited years after their stroke, and whether this fatigue is a function of other issues. Future systematic reviews could address this issue by conducting sensitivity analyses comparing studies that include participants many years after their stroke with those including participants immediately after their stroke."

**Comment**: International recommendations regarding outcome measures related to, and time points for measuring, post-stroke fatigue in research studies on recovery after stroke would probably reduce the heterogeneity of studies and facilitate further summaries and updates. You could possibly include that post-stroke fatigue might be an issue for future Stroke Recovery and Rehabilitation Roundtable (SRRR) work.

**Reply**:

 Thank you for this suggestion. We have made a comment about this being an important area for future research but do not want to identify an individual group or organization to take this forward. We added: "In addition, harmonisaton of studies requires standard international guidelines regarding outcome measurements and time points for measuring PSF in a standard way to create homogenous and collective body of evidence.".

**Comment**: You have a separate paragraph regarding limitations. Have you thought about including a sentence related to strengths of your study, in a paragraph in close proximity to "Limitations"?

**Reply**:Thank you for this comment. We have changed the heading to 'Limitations and Strengths' and added three strengths of the review in this section: A. Public and open sharing of our methods so that anyone can update our review, B. Utilizing automation tools such as Rayyan and RobotReviewer to save time and resources and C. The fact that this was A multi-disciplinary review team.
